# Choice and Duration of Antimicrobial Therapy for Neonatal Sepsis and Meningitis

**DOI:** 10.1155/2011/712150

**Published:** 2011-11-20

**Authors:** Sindhu Sivanandan, Amuchou S. Soraisham, Kamala Swarnam

**Affiliations:** ^1^Division of Neonatology, Department of Pediatrics, University of Calgary, Calgary, AB, Canada T2N 1N4; ^2^Institute for Maternal and Child Health, University of Calgary, Calgary, AB, Canada T2N 4N1

## Abstract

Neonatal sepsis is associated with increased mortality and morbidity including neurodevelopmental impairment and prolonged hospital stay. Signs and symptoms of sepsis are nonspecific, and empiric antimicrobial therapy is promptly initiated after obtaining appropriate cultures. However, many preterm and low birth weight infants who do not have infection receive antimicrobial agents during hospital stay. Prolonged and unnecessary use of antimicrobial agents is associated with deleterious effects on the host and the environment. Traditionally, the choice of antimicrobial agents is based on the local policy, and the duration of therapy is decided by the treating physician based on clinical symptoms and blood culture results. In this paper, we discuss briefly the causative organism of neonatal sepsis in both the developed and developing countries. We review the evidence for appropriate choice of empiric antimicrobial agents and optimal duration of therapy in neonates with suspected sepsis, culture-proven sepsis, and meningitis. Moreover, there is significant similarity between the causative organisms for early- and late-onset sepsis in developing countries. The choice of antibiotic described in this paper may be more applicable in developed countries.

## 1. Introduction

Neonatal sepsis is a clinical syndrome characterized by systemic signs of infection and accompanied by bacteremia in the first month of life [1]. Sepsis occurring in the first 72 hours of life is defined as early-onset sepsis (EOS) [2] and that occurring beyond 72 hours as late-onset sepsis (LOS). Neonatal sepsis is associated with significant morbidity and mortality justifying prompt initiation of appropriate empirical antibiotic therapy. Knowledge of both the common pathogens causing septicemia in neonates and their antimicrobial susceptibility is essential in order to select appropriate antimicrobial treatment. Antimicrobial susceptibility patterns of pathogens vary geographically and are temporally dependent on local pathogens and patterns of antibiotic use. The aim of this paper is to review the literature regarding the appropriate choice and duration of antimicrobial therapy for suspected sepsis, culture-proven sepsis, and meningitis during neonatal period. We would like to focus mainly on the treatment of bacterial infection.

## 2. Microbiology of Neonatal Sepsis 

The majority of cases of EOS result from vertical transmission of bacteria from mother to the neonate during the intrapartum period. LOS is due to the horizontal transmission of pathogens from the environment or the hands of the caregiver. The pattern of bacterial pathogen responsible for neonatal sepsis has changed with time and varies from place to place. There is a difference in the causative organisms for neonatal sepsis between the developed and developing countries [2–9]. Within developing countries, there are regional variations in the spectrum of organisms causing neonatal sepsis. 

 In United States, the National Institute of Child Health and Development (NICHD) reported that the common pathogens causing EOS are group B *Streptococcus *(GBS) and *Escherichia coli *[2]. GBS remains the most frequent pathogen in term infants, and *E. coli *the most significant pathogen in preterm infants with EOS [2]. Similarly, a study from the United Kingdom has reported GBS to be the most frequent pathogen (31%) followed by coagulase-negative *Staphylococcus *(CoNS), nonpyogenic *streptococci* and *E. coli* [3]. 

In the developed countries, Gram-positive organisms account for about 70% of all LOS. The common pathogens causing LOS in very low birth weight (VLBW) infants include CoNS followed by *Staphylococcus aureus*, *Enterococcus *spp., and GBS [3–5]. About 18–20% of late-onset sepsis is caused by Gram-negative organisms especially *Enterobacteriaceae *spp. and *E. coli*. About 12% of LOS sepsis is caused by fungi especially *Candida *species [4].

In the developing world, *E. coli, Klebsiella species*, and* S. aureus* are the most common pathogens of EOS, whereas *S. aureus*, *Streptococcus pneumonia*, and* Streptococcus pyogenes *are the most commonly reported organisms in LOS [6, 7]. According to the National Neonatal Perinatal Database of India, *Klebsiella pneumonia*, *Staphylococcus aureus*, and *E. coli* are the three most common organisms causing neonatal sepsis both in hospital and community [8]. Moreover, the causative organisms of EOS and LOS sepsis are similar especially in hospital setting in developing country [9].

## 3. Empirical Antimicrobial Therapy for Neonatal Sepsis

Although isolation of a pathogen in culture is a prerequisite for proven bacterial sepsis, culture results take at least 48–72 hours to be reported. The early and appropriate initiation of antimicrobial agents in high-risk neonates before the result of blood culture susceptibility is defined as “empirical antibiotic therapy.” Most infants admitted to the neonatal intensive care units (NICUs) receive empirical antibiotics when in fact the incidence of culture-proven EOS is only between 1 and 4.6 cases per 1000 live births [10, 11]. The clinical manifestations of neonatal sepsis are nonspecific, and the fear of missing the diagnosis is high because of the increased morbidity and mortality associated with sepsis. One study noted that the ratio of noninfected to blood-culture-positive neonates treated with antibiotics was between 15 : 1 and 28 : 1 [12]. Early and prompt empirical antibiotic therapy, based on risk-factor-driven decision in EOS [13, 14] and clinical symptoms in LOS [15], have been shown to reduce mortality in neonates. The risk factors for EOS include clinical chorioamnionitis, maternal intrapartum fever (>38.0°C), delivery at <37 weeks, rupture of membranes (ROM) >18 hours before delivery, maternal GBS colonization, previous infant with GBS infection, GBS bacteriuria, and inadequate intrapartum antibiotic prophylaxis [16]. Among infants who are discharged home, new onset of fever, cough, fast or difficult breathing, poor feeding, lethargy, and convulsions are indicators of sepsis and warrant initiation of appropriate antibiotic therapy [17]. 

### 3.1. Empirical Antimicrobial Regimen for Early-Onset Sepsis

Treatment of neonates with suspected sepsis or meningitis should commence as soon as appropriate cultures and intravenous access can be obtained. The initial choice of antimicrobial agents for empirical treatment is dependent on the knowledge of the probable pathogens based on the perinatal history, including any maternal symptoms, cultures, or instrumentation and susceptibility pattern of the organisms [18]. 

Prospective randomized controlled trials (RCTs) on the appropriate choice of antimicrobial therapy in the neonate with suspected sepsis are limited [19].  Table 1 summarizes the RCTs on appropriate empirical antimicrobial treatment options in EOS. Cochrane meta-analysis of 2 RCTs by Snelling et al. [19] and Miall-Allen et al. [20] that compared monotherapy to combination antimicrobial therapy failed to show that one regime was superior to the other [13]. There was no significant difference in mortality, treatment failure, or bacteriological resistance. These two trials had small sample size and were done prior to 1990, and the antibiotics used in the study (ceftazidime, timentin, and piperacillin) are no longer used in empirical therapy today. This meta-analysis excluded 12 trials comparing various combinations of antibiotic therapy due to two reasons: the study population enrolled neonates older than 48 hours or even older children and a distinction between EOS, and LOS was not made. 

 In an open label cluster randomized study, Metsvaht et al. [21] from Estonia compared the clinical efficacy of ampicillin versus penicillin G both combined with gentamicin in the empirical treatment of neonates (*n* = 283) at risk of EOS. Incidence of proven EOS was 4.9% in this study. The clinical failure rate was not different between the two groups (14.1% versus 14.2%).

In the NICHD neonatal network study [2], all GBS isolates were sensitive to penicillin, ampicillin, and vancomycin. Among *E. coli *isolates, 96% were sensitive to gentamicin and cephalosporin, but up to 78% were resistant to ampicillin. The national laboratory surveillance data from England and Wales showed that nearly all (94%) of the early-onset isolates (GBS, CoNS, nonpyogenic *streptococci*, *E. coli*, etc.) were sensitive to an antibiotic regimen of penicillin + gentamicin and 100% were sensitive to the combination of amoxicillin + cefotaxime [3]. 

Based on the common antibiotic susceptibilities of the predominant organism causing EOS, the recommended initial empiric therapy for a neonate with suspected bacterial sepsis and/or meningitis includes ampicillin and an aminoglycoside [22, 23]. This combination expands the antimicrobial spectrum and also offers synergistic bacterial killing. The other advantages are low cost and low rates of emergence of bacterial resistance [24].

However, in developing countries where the causative organisms of EOS are different from the developed countries, the above combination of ampicillin and gentamicin may not be the best empirical antibiotic of choice. Recently, Lubell et al. [25] reviewed the literature regarding the antibiotic susceptibility patterns of community-acquired pathogens causing neonatal sepsis in sub-Saharan Africa and Asia. The two common pathogens, *S. aureus *and* Klebsiella *spp., exhibited high rates of resistance to almost all commonly used antibiotics (ampicillin, ceftriaxone, chloramphenicol, cotrimoxazole, macrolides, and gentamicin). Only *Streptococcus pneumoniae* exhibited good susceptibility to all drugs other than cotrimoxazole. The available studies from developing world focus mainly on community-acquired infections, and segregation between EOS and LOS is often not available. More than 99% of neonatal deaths occur in the developing world, and a quarter of these deaths are attributed to neonatal sepsis [26]. Insufficient knowledge about the appropriate antibiotic choice and the emerging resistance to commonly prescribed antimicrobials in neonatal sepsis will hamper the ability to successfully treat this condition in the developing world. 

In summary, based on current available evidence, the combination of ampicillin and gentamicin is an appropriate choice for empirical therapy of EOS in neonates, where GBS and *E. coli* continue to be the predominant organisms. In developing countries, empiric antibiotic therapy should be based individualized for each hospital or region.

### 3.2. Empirical Antimicrobial Therapy for Suspected Late-Onset Sepsis

As discussed earlier, CoNS is the most common pathogen in LOS followed by *S. aureus*, *Enterococcus *spp., and GBS; Gram-negative organisms account for 18–20% of LOS [3–5]. The empirical antimicrobial therapy for LOS should cover both Gram-positive and Gram-negative organisms. In the study from the United Kingdom, more than 95% of organisms causing LOS were susceptible to gentamicin with either flucloxacillin or amoxicillin and amoxicillin with cefotaxime, but only 79% were susceptible to cefotaxime monotherapy [3]. 

In the developed countries, where CoNS is the predominant nosocomial pathogen and where resistance of these isolates to penicillin, semisynthetic penicillin, and gentamicin are common, experts recommend the use of vancomycin as empirical therapy [24]. Of the 18 participating NICUs in Australasian study group for neonatal infection, nine units used vancomycin and an aminoglycoside as the first-line empirical treatment for LOS [5]. Their mortality from CoNS sepsis was no different from the remaining units that used ampicillin or flucloxacillin together with an aminoglycoside. The authors suggest that overvigorous attempts to reduce the incidence of CoNS infections using prophylactic antibiotics are not advisable as the majority of these infections are relatively benign. 

To reduce the growing emergence of resistant strains due to injudicious use of vancomycin, Karlowicz et al. [27] studied the impact of changing empiric antibiotic therapy for LOS from vancomycin and cefotaxime to oxacillin and gentamicin. There was no impact on the frequency of fulminant sepsis due to CoNS even though 85% of CoNS isolates were resistant to either oxacillin or gentamicin. Most cases of fulminant LOS were caused by Gram-negative organisms rather than CoNS. Similar findings were reported by Stoll et al. [4]. 

In developing nations, LOS is complicated by a higher percentage of Gram-negative bacteria and greater antimicrobial resistance among the organisms. Zaidi et al. [28] reported that the rates of neonatal sepsis were 3–20 times higher among hospitalized infants in developing countries compared to developed nations. *Klebsiella pneumoniae*, other Gram-negative rods (*E. coli*, *Pseudomonas *spp., and *Acinetobacter *spp.), and *S. aureus *were the major pathogens among 11471 bloodstream isolates. About 70% of these isolates may not be covered by the empiric regimen of ampicillin and gentamicin. About half of *E. coli* and *Klebsiella* isolates were resistant to cefotaxime, a commonly used second-line antibiotic. The important concern is the high proportion of methicillin-resistant *S. aureus *(MRSA) strains in many areas, especially south Asia where 56% of all isolates were reported to be methicillin resistant. 

The Cochrane review of empirical antibiotic therapy in LOS [29] included one study by Miall-Allen et al. [20] that compared timentin (ticarcillin and clavulanic acid) monotherapy with a combination of flucloxacillin and gentamicin in 28 neonates with suspected LOS. Although the study did not meet the criteria for methodological quality, there was no difference in outcome (mortality/treatment failure) between the two groups. 

Empiric antifungal therapy should be considered if the infants have central vascular access, an endotracheal tube, thrombocytopenia (<100,000/mm^3^), exposure to broad-spectrum cephalosporins or carbapenem, and gestational age less than 28 weeks [30, 31]. Amphotericin B should be chosen for empiric therapy, and fluconazole should be reserved for prophylaxis. 

In summary, there is inadequate evidence from randomized trials in favor of any particular antimicrobial regimen for the empirical treatment of suspected LOS. An acceptable approach would be to start with cloxacillin and gentamicin as initial antibiotics for LOS in a stable neonate. Vancomycin and third-generation cephalosporin (e.g., cefotaxime) should be considered for LOS in a neonate presenting with cardiorespiratory instability and in areas where MRSA is prevalent. The dangers of starting vancomycin as the initial therapy in all infants include the risk of emergence of vancomycin-resistant *enterococci *and its overuse in cases where CoNS isolates represent mere contaminants.

## 4. Duration of Empirical Antimicrobial Therapy

There is a wide variation between centers regarding the appropriate duration of empiric antibiotics for suspected EOS when blood cultures are negative [32]. This is more pertinent for preterm infants (gestational age <34 weeks and/or <1500 grams) as they are considered to be at high risk for sepsis, and the usual practice is to draw appropriate cultures and start antibiotics. The recommendations for treatment of infants with suspected sepsis but who have negative cultures are not based on strong evidence. The standard practice is to discontinue antibiotics as soon as blood cultures are confirmed negative (48–72 hours) and there are no clinical or hematologic signs of infection [32–34]. This recommendation is originally based on Pichichero's study [35] who reported that 96% of bacteremic cultures drawn prior to antibiotic therapy are positive by 48 hours and 98% are positive by 72 hours. 

With the use of computer-assisted automated culture media (the ESP blood culture system), Garcia-Prats et al. [36] reported that 89% of blood cultures of potentially septic neonates (term and preterm) become positive by 36 hours and 94% by 48 hours. The time to blood culture positivity was not affected by prior antibiotic therapy. The authors concluded that, in term asymptomatic infants treated with antibiotics based on maternal risk factors, the duration can be reduced to 24–36 hours where reliable and speedier blood culture results are available.

There is a lack of well-designed adequately powered trials evaluating the appropriate duration of empirical antimicrobial therapy in blood-culture-negative sepsis. The only pilot study by Saini et al. [37] randomized 52 infants (>30 weeks gestation and >1000 g at birth), with culture-negative probable sepsis to either short-course (48–96 hours) or long-course (7 days) antibiotic therapy. The randomization was done 48–72 hours after enrollment. Infants with confirmed sepsis and those who had persisting clinical symptoms were excluded. The choice of antibiotic therapy (cephalosporin, amikacin, and cloxacillin) was made by the treating physician. There was no difference in treatment failure (defined as reappearance of signs of sepsis within 15 days of stopping antibiotics, supported by laboratory evidence) rate between the 2 groups. The limitations of this study include its small sample size, lack of segregation between early- and late-onset sepsis, and the exclusion of smaller babies. 

Cordero and Ayers [38] reported that it is probably safe to discontinue empiric antibiotics when blood cultures are negative in asymptomatic extremely low birth weight (ELBW) neonates. They noted that ELBW infants treated with a shorter course (≤3 days) for suspected sepsis had lesser exposure to a third antimicrobial (vancomycin, amphotericin, or oxacillin) and lesser antibiotic days compared to a longer course (≥7 days).

Although prospective studies are limited, it seems reasonable to conclude that the duration of empirical antimicrobial therapy should be 48–72 hours pending culture results for suspected neonatal sepsis. A symptomatic baby can have a false-negative blood culture if antibiotics are given prenatally to the mother or if the blood sample is collected improperly. Hence, antibiotics should be continued for symptomatic infants and those with positive blood culture.

## 5. Role of C-Reactive Protein in Guiding the Duration of Antimicrobial Therapy 

C-reactive protein (CRP) is an excellent marker for established neonatal bacterial infections. However, it is not useful for early diagnosis because levels are elevated only in 35% to 65% of neonates at the onset of illness [39]. Several studies have evaluated the role of serial CRP measurement as a guide to the duration of antibiotic therapy both in developed and developing countries [40–44]. Serial CRP values taken 24–48 h after the onset of symptoms have an improved sensitivity and specificity when compared with single CRP values at presentation for diagnosis of sepsis. Two consecutive CRP levels <10 mg/L 24 hours apart, 8–48 hours after presentation, have a negative predictive value for sepsis of 99% [40]. 

Philip and Mills [41] suggested that normalization of CRP levels can be considered as a criterion for the discontinuation of antibiotic therapy to minimize antibiotic exposure and shorten hospital stay. In a prospective study, Ehl et al. [42] observed that CRP levels of <10 mg/L determined 24 hours after beginning antibiotic treatment correctly identified 120 of 121 infants as not needing further antibiotics. Jaswal et al. [43] reported a 100% negative predictive value with no relapse following discontinuation of antibiotic treatment after normalization of CRP levels. Contrary to the previous studies, Al-Zwaini et al. [44] reported CRP to be a poor guide for the duration of treatment in neonatal septicemia, with modest sensitivity and negative predictive value (78% and 86%, resp.) at 48 hours following initiation of antibiotics. 

The physicians are in constant dilemma regarding the duration of antibiotic therapy in neonates with likely infection who have nonspecific clinical symptoms and negative blood cultures or those infants who undergo incomplete sepsis workup. In such cases, Squire et al. [45] suggested that, by augmenting the sepsis evaluation with a negative CRP, total antibiotic use may be reduced by up to 20% for the entire nursery. However, previous studies that showed CRP as a good guide excluded high-risk infants with central lines, mechanical ventilation, postsurgery, meningitis, birth asphyxia [42], and those with positive initial CRP [40]. Hence, the usefulness of CRP in guiding decisions regarding the duration of antibiotics might be valid only in selected subset of neonates.

## 6. Hazards of Prolonged Empirical Antimicrobial Therapy

Optimal duration of empiric antimicrobial use decreases the development of antimicrobial resistance, prevents unwanted changes in flora found in the NICU, and minimizes unnecessary expenses for infants who have negative blood cultures [46, 47]. About half of all ELBW infants receive empirical antibiotics for prolonged periods (>3–5 days) despite negative blood cultures [47, 48]. Prolonged duration of initial empirical antibiotic therapy is associated with an increased risk of necrotizing enterocolitis and death in ELBW infants [48]. Clark et al. [49] observed that selection of cefotaxime (a third-generation cephalosporin) instead of gentamicin for the first 3 postnatal days is associated with higher mortality rate, even for the most preterm infants. Other adverse effects of prolonged empirical antibiotic therapy include increased risk of neonatal candidiasis with the use of cephalosporins [50, 51] and alteration of gut microflora [52]. Hence, it seems prudent to restrict the duration of empirical antibiotic therapy to <3 days when blood cultures are sterile and baby is asymptomatic.

## 7. Duration of Antimicrobial Therapy for Proven Bacterial Sepsis without Meningitis

The duration of antimicrobial therapy for culture-proven sepsis depends on the initial response to the appropriate antimicrobial agent. In the chapter on bacterial sepsis and meningitis, Klein recommend 10 days of therapy for culture-proven sepsis with minimal or absent focal infection [22]. However, there is a paucity of RCTs evaluating the rationale and safety of the appropriate duration of therapy [53–55].  Table 2 describes the RCTs evaluating shorter versus longer courses of antibiotic therapy in neonatal pneumonia and culture-proven sepsis. 

Engle et al. [53] randomized cases of neonatal pneumonia to either 4-day or 7-day course of antibiotics. Randomization was done on day 4 of antibiotic therapy if the infants were completely asymptomatic for at least 48 hours. Infants in the 4-day course were observed for 24 hours after stopping antibiotics and were reassessed as outpatients within 2-3 days following discharge. The success of therapy, as defined by infants doing well after discharge and no need for rehospitalization for sepsis or pneumonia, was similar in both the groups, and the length of hospitalization was shorter in 4-day therapy.

Chowdhary et al. [54] compared the effectiveness of 7-day versus 14-day antibiotic therapy in 69 infants with blood culture-proven bacterial sepsis (without meningitis and deep seated infections). Randomization to either 7-day or 14-day therapy was performed on day 7 of antibiotics if the infant had clinical remission by day 5. Blood culture was repeated 24 hours after antibiotic completion, and infants were observed in the hospital for at least 72 hours and followed up for 28 days. There was a trend towards more treatment failures (defined as a positive blood culture, or clinical signs) in 7-day group as compared to 14-day group (5 infants versus 1 infant, *P* = 0.19). Subgroup analysis revealed that treatment failures occurred in subjects with *S. aureus* infection receiving 7-day course (4 infants). *S. aureus* accounted for 20% positive cultures. The authors concluded that neonates with *S. aureus* infection may require 14 days of antibiotic therapy.

Gathwala et al. [55] compared the effectiveness of a 10-day versus 14-day course of antibiotic therapy in blood culture-proven neonatal sepsis. Sixty infants were randomized on day 7 of antibiotic therapy, if they were in clinical remission with negative C-reactive protein (CRP). Cefotaxime and amikacin were used in all infants. The most common organism causing sepsis was *Pseudomonas aeruginosa* in both of the groups, followed by *Acinetobacter* and *Klebsiella*. The incidence of CoNS and *S. aureus* was 3% and 8%, respectively. The primary outcome of treatment failure within 28 days occurred in one infant in each group. The duration of hospital stay was significantly shorter in the 10-day course as compared with the 14-day course (13 ± 1.7 days versus 17 ± 2.2 days). They concluded that 10-day antibiotic therapy is as effective as 14-day therapy in blood-culture-proven neonatal sepsis, if the infant has achieved clinical remission by day 7 of therapy. 

In the above 3 trials, the participants were ≥32 weeks or ≥1500 grams and were in clinical remission at the time of randomization. There is limited evidence for infants with younger gestational age (<32 weeks), who are at the highest risk for sepsis. In other words, the shorter duration seemed effective only in selected subset of neonates who were at least 32 weeks and above and showed good initial response to antibiotics (milder disease).

In summary, pending further evidence, it is reasonable to treat for 10–14 days with appropriate antimicrobial agents in infants with blood-culture-proven sepsis. However, in selected situations (neonates ≥32 weeks gestation and ≥1500 grams, who become asymptomatic within 5 days of appropriate therapy), we can consider stopping antibiotics at 7–10 days, provided appropriate followup can be ensured.

## 8. Antimicrobial Choice and Duration of Therapy for Neonatal Meningitis

Decisions on the choice of a specific antimicrobial agent are based on knowledge of its activity against the causative pathogen and relative penetration into cerebrospinal fluid (CSF) in the presence of meningeal inflammation. In 2004, Infectious Disease Society of America published practice guidelines for treatment of meningitis [56]. When bacterial meningitis is suspected as part of EOS, ampicillin with either an aminoglycoside or cefotaxime is commonly recommended as initial empirical therapy to cover GBS,* E. coli*, *Listeria monocytogenes*, and* Klebsiella *species [56]. For neonates with late-onset meningitis, a regimen containing an antistaphylococcal antibiotic, such as nafcillin or vancomycin, plus cefotaxime or ceftazidime with or without an aminoglycoside is recommended [57].

 The duration of antimicrobial therapy in the patient with bacterial meningitis has often been based more on tradition than on evidence-based data. GBS meningitis is usually treated for 14 to 21 days, assuming prompt eradication of bacteria from the CSF. For uncomplicated neonatal meningitis caused by Gram-negative bacteria, a minimum of 21 days is recommended [56].

However, these guidelines are not standardized and the duration of therapy may need to be individualized on the basis of the patient's clinical response. Failure to achieve CSF sterilization or persistence of symptoms should prompt the clinicians to look for possible complications such as brain abscess, ventriculitis, or subdural empyema. Infants with intracranial abscesses should be treated with a combination of surgical aspiration or drainage of the abscess plus antimicrobial therapy for 4–6 weeks [58]. Early neuroimaging by ultrasonography, magnetic resonance imaging (MRI), or computed tomography (CT) is indicated to assess the need for surgical intervention. Imaging should be repeated even after the antibiotic therapy has been completed, as there are reports of abscesses being identified weeks after the initiation of antibiotic therapy. Despite adequate antimicrobial therapy for 21 days or more, relapses may occur in meningitis caused by Gram-negative enteric bacilli [59]. 

A systematic review of short-course antibiotic therapy for meningitis in children (3 weeks to 16 years) showed that there was no difference between short-course (4–7 days) and long-course (7–14 days) treatment (intravenous ceftriaxone) regarding end of therapy clinical success, long term neurological complications, hearing impairment, or secondary nosocomial infections [60]. The duration of hospitalization was lesser with short-course regimen. The antibiotic used was ceftriaxone in all the 5 studies included in the systematic review. The CSF concentration of ceftriaxone is many times higher than the minimum inhibitory concentration for most causative pathogens [61]. In neonates, cefotaxime is often preferred over ceftriaxone (particularly for those who have hyperbilirubinemia) because ceftriaxone can displace bilirubin from albumin binding sites. Moreover, the patients enrolled in the above trials were at least 3 months old (except for one study that enrolled from 3 weeks onwards). Hence, the result of the systematic review cannot be extrapolated to neonates who constitute a different subset by virtue of their compromised immune status. Finally, the shorter courses may not be applicable for patients who receive adjuvant therapy with dexamethasone. The potential suppression of immune responses caused by dexamethasone may warrant a relatively long duration of antimicrobial therapy for optimal microbiological outcomes [60]. 

In one RCT, there was no clinical benefit of adjuvant dexamethasone therapy in 52 full-term neonates with bacterial meningitis [62]. However, a prospective nonrandomized study from Nigeria reported that dexamethasone significantly decreased the mortality and neurological sequelae in neonatal bacterial meningitis. At present, there is insufficient data to make a recommendation on the use of adjunctive dexamethasone in neonatal bacterial meningitis [56].

Repeat lumbar puncture to document CSF sterilization and improvement of CSF parameters is not indicated routinely [56]. However, it should be done in all patients who have not responded clinically after 48 hours of appropriate antimicrobial therapy. Neonates with meningitis due to Gram-negative bacilli should undergo repeated lumbar punctures to document CSF sterilization, because the duration of antimicrobial therapy is determined, in part, by the result. Some authors recommend CSF examination at the completion of therapy for all neonates to establish whether additional treatment is required because of the unpredictable clinical course of illness and the unreliability of the clinical examination in assessment of response to treatment in neonates [57].

 In summary, combination of ampicillin and cefotaxime or ampicillin and aminoglycoside is appropriate for treatment of suspected early-onset neonatal meningitis. For suspected late-onset meningitis, a combination of vancomycin plus a third-generation cephalosporin is recommended while awaiting CSF culture and susceptibility results. The duration of antimicrobial therapy for neonatal meningitis should be 14 to 21 days for GBS, ≥21 days for *L. monocytogenes* meningitis, and minimum of 21 days for Gram-negative meningitis.

## 9. Conclusion

The choice of antibiotics should be based on the causative organisms and the patterns of antibiotic susceptibility. The combination of ampicillin and gentamicin is an appropriate choice for empirical therapy of neonatal EOS in developed countries where GBS and *E. coli* continue to be the predominant organisms. For LOS, starting cloxacillin and gentamicin may be appropriate in a stable neonate. Vancomycin and cefotaxime should be considered in sick neonates with cardiorespiratory compromise and in areas where the MRSA is prevalent. In developing nations where the causative organisms and pattern of antibiotic susceptibility are different, the choice of antimicrobial therapy should be modified based on local prevalence. 

The duration of empirical antibiotic therapy in neonates should be 48–72 hours pending culture results for suspected sepsis. Until further evidence, the current recommendation of 10–14 days of antimicrobial treatment is appropriate for blood-culture-positive sepsis without meningitis. Neonates with bacterial meningitis require longer duration based on the organism isolated. Repeat lumbar puncture to document CSF sterilization is important because of unpredictable clinical course in neonates. Above all a disciplined approach consisting of a thorough physical examination and evaluation of clinical response to treatment are important in tailoring appropriate dose and duration of antibiotics in neonates with suspected or proven sepsis. 

## Figures and Tables

**Table 1:** Randomized controlled trials on empirical antimicrobial therapy for suspected early-onset sepsis.

Author	Population	Antibiotics used	Duration of treatment	Outcomes	Result
Snelling et al., 1983 [19]	55 neonates <48 hours old with suspected sepsis	Ceftazidime (*n* = 31) versus gentamicin plus benzyl penicillin (*n* = 24)	48 hours if culture was sterile. 7 days if culture was positive or infant symptomatic	Mortality, treatment failure, bacteriological resistance	Ceftazidime is good as penicillin and gentamicin

Miall-Allen et al., 1988 [20]	72 neonates <48 hours old with suspected sepsis	Timentin (ticarcillin + clavulanic acid) (*n* = 32) versus piperacillin ± gentamicin (*n* = 40)	Variable based on clinical and bacterial remission. Maximum 10 days	Mortality, treatment failure, bacteriological resistance	No difference in mortality or treatment failure

Metsvaht et al., 2010 [21]	283 neonates <72 hours old with suspected sepsis	Ampicillin + gentamicin (*n* = 142) versus penicillin + gentamicin (*n* = 141)	Not specified	Treatment failure (defined as need for a change in the initial antibiotic regimen within 72 h and/or 7-day all-cause mortality)	No difference in treatment failure or mortality rate between the two regimens

**Table 2:** Randomized controlled trials of short- versus long-course antibiotic therapy in neonatal bacterial sepsis/pneumonia.

Author	Population	Antibiotics used	Duration of treatment	Outcomes	Conclusion
Engle et al., 2000 [53]	Term and near-term neonates with pneumonia. Excluded babies with meconium stained amniotic fluid and O_2_ requirement for >8 hours.	Ampicillin and gentamicin	4 days (*n* = 35) versus 7 days (*n* = 38)	Success defined as neonates doing well after discharge and no need for rehospitalization for sepsis or pneumonia	The success rate for therapy was similar between the two groups

Chowdhary et al., 2006 [54]	≥32 weeks and >1500 grams with positive blood culture. Excluded deep seated infections and meningitis	Not specified	7 days (*n* = 34) versus 14 days (*n* = 35)	Treatment failure within 28 days	There was a trend towards more treatment failures in 7-day group as compared to 14-day group (5 infants versus 1 infant, *P* = 0.19)

Gathwala et al., 2010 [55]	Infants ≥32 weeks and >1500 grams with positive blood culture. Excluded deep seated infections and meningitis	Cefotaxime and amikacin	10 days (*n* = 30) versus 14 days (*n* = 30)	Treatment failure within 28 days	10-day course was as effective as 14-day course in blood-culture-proven neonatal

## References

[1] Klein JO (1990). Bacteriology of neonatal sepsis. *Pediatric Infectious Disease Journal*.

[2] Stoll BJ, Hansen NI, Sánchez PJ (2011). Early onset neonatal sepsis: the burden of group B streptococcal and E. coli disease continues. *Pediatrics*.

[3] Muller-Pebody B, Johnson AP, Heath PT, Gilbert RE, Henderson KL, Sharland M (2011). Empirical treatment of neonatal sepsis: are the current guidelines adequate?. *Archives of Disease in Childhood: Fetal and Neonatal Edition*.

[4] Stoll BJ, Hansen N, Fanaroff AA (2002). Late-onset sepsis in very low birth weight neonates: the experience of the NICHD Neonatal Research Network. *Pediatrics*.

[5] Isaacs D (2003). A ten year, multicentre study of coagulase negative staphylococcal infections in Australasian neonatal units. *Archives of Disease in Childhood: Fetal and Neonatal Edition*.

[6] Zaidi AKM, Thaver D, Ali SA, Khan TA (2009). Pathogens associated with sepsis in newborns and young infants in developing countries. *Pediatric Infectious Disease Journal*.

[7] Mulholland K, Margolis P, Mason K (1999). Bacterial etiology of serious infections in young infants in developing countries: results of a multicenter study. *Pediatric Infectious Disease Journal*.

[8] Report of the National Neonatal Perinatal Database (National Neonatology Forum).

[9] Sundaram V, Kumar P, Dutta S (2009). Blood culture confirmed bacterial sepsis in neonates in a north Indian tertiary care center: changes over the last decade. *Japanese Journal of Infectious Diseases*.

[10] Baltimore RS, Huie SM, Meek JI, Schuchat A, O’Brien KL (2001). Early-onset neonatal sepsis in the era of group B streptococcal prevention. *Pediatrics*.

[11] Stoll BJ, Hansen N, Fanaroff AA (2002). Changes in pathogens causing early-onset sepsis in very-low-birth-weight infants. *The New England Journal of Medicine*.

[12] Hammerschlag MR, Klein JO, Herschel M, Chen FC, Fermin R (1977). Patterns of use of antibiotics in two newborn nurseries. *The New England Journal of Medicine*.

[13] Mtitimila EI, Cooke RW (2004). Antibiotic regimens for suspected early neonatal sepsis. *Cochrane Database of Systematic Reviews*.

[14] Dawodu AH, Effiong CE (1985). Neonatal mortality: effects of selective pediatric interventions. *Pediatrics*.

[15] Margolis P, Mulholland EK, Harrell F (1999). Clinical prediction of serious bacterial infections in young infants in developing countries. *Pediatric Infectious Disease Journal*.

[16] Verani JR, McGee L, Schrag SJ (2010). Prevention of perinatal group B streptococcal disease revised guidelines from CDC. *Morbidity and Mortality Weekly Report*.

[17] (1999). Methodology for a multicenter study of serious infections in young infants in developing countries. *Pediatric Infectious Disease Journal*.

[18] Gerdes JS (2004). Diagnosis and management of bacterial infections in the neonate. *Pediatric Clinics of North America*.

[19] Snelling S, Hart CA, Cooke RWI (1983). Ceftazidime or gentamicin plus benzylpenicillin in neonates less than forty-eight hours old. *Journal of Antimicrobial Chemotherapy*.

[20] Miall-Allen VM, Whitelaw AGL, Darrell JH (1988). Ticarcillin plus clavulanic acid (Timentin) compared with standard antibiotic regimes in the treatment of early and late neonatal infections. *British Journal of Clinical Practice*.

[21] Metsvaht T, Ilmoja ML, Parm Ü, Maipuu L, Merila M, Lutsar I (2010). Comparison of ampicillin plus gentamicin vs. penicillin plus gentamicin in empiric treatment of neonates at risk of early onset sepsis. *Acta Paediatrica, International Journal of Paediatrics*.

[22] Klein JO, Remington JS, Klein JO (2006). Bacterial sepsis and meningitis. *Infectious Disaeses of the Fetus and Newborn Infant*.

[23] Pickering LK, Baker CJ, Kimberlin DW, Long SS, American Academy of Pediatrics (2009). Group B streptococcal infections. *Red Book: 2009 Report of the Committee on Infectious Diseases*.

[24] Baltimore RS (2003). Neonatal Sepsis: epidemiology and Management. *Pediatric Drugs*.

[25] Lubell Y, Ashley EA, Turner C, Turner P, White NJ (2011). Susceptibility of community-acquired pathogens to antibiotics in Africa and Asia in neonates—an alarmingly short review. *Tropical Medicine and International Health*.

[26] Lawn JE, Cousens S, Zupan J (2005). 4 Million neonatal deaths: when? Where? Why?. *Lancet*.

[27] Karlowicz MG, Buescher ES, Surka AE (2000). Fulminant late-onset sepsis in a neonatal intensive care unit, 1988–1997, and the impact of avoiding empiric vancomycin therapy. *Pediatrics*.

[28] Zaidi AKM, Huskins WC, Thaver D, Bhutta ZA, Abbas Z, Goldmann DA (2005). Hospital-acquired neonatal infections in developing countries. *Lancet*.

[29] Gordon A, Jeffery HE (2005). Antibiotic regimens for suspected late onset sepsis in newborn infants. *Cochrane Database of Systematic Reviews*.

[30] Benjamin DK, DeLong ER, Steinbach WJ, Cotton CM, Walsh TJ, Clark RH (2003). Empirical therapy for neonatal candidemia in very low birth weight infants. *Pediatrics*.

[31] Kaufman D, Fairchild KD (2004). Clinical microbiology of bacterial and fungal sepsis in very-low-birth-weight infants. *Clinical Microbiology Reviews*.

[32] Stoll BJ, Gordon T, Korones SB (1996). Early-onset sepsis in very low birth weight neonates: a report from the national institute of child health and human development neonatal research network. *Journal of Pediatrics*.

[33] Kaftan H, Kinney JS (1998). Early onset neonatal bacterial infections. *Seminars in Perinatology*.

[34] Cordero L (1999). Bloodstream infections in a neonatal intensive-care unit: 12 years’ experience with an antibiotic control program. *Infection Control and Hospital Epidemiology*.

[35] Pichichero ME, Todd JK (1979). Detection of neonatal bacteremia. *Journal of Pediatrics*.

[36] Garcia-Prats JA, Cooper TR, Schneider VF, Stager CE, Hansen TN (2000). Rapid detection of microorganisms in blood cultures of newborn infants utilizing an automated blood culture system. *Pediatrics*.

[37] Saini SS, Dutta S, Ray P, Narang A (2011). Short course versus 7-day course of intravenous antibiotics for probable neonatal septicemia: a pilot, open-label, randomized controlled trial. *Indian Pediatrics*.

[38] Cordero L, Ayers LW (2003). Duration of empiric antibiotics for suspected early-onset sepsis in extremely low birth weight infants. *Infection Control and Hospital Epidemiology*.

[39] Benitz WE, Han MY, Madan A, Ramachandra P (1998). Serial serum C-reactive protein levels in the diagnosis of neonatal infection. *Pediatrics*.

[40] Bomela HN, Ballot DE, Cory BJ, Cooper PA (2000). Use of C-reactive protein to guide duration of empiric antibiotic therapy in suspected early neonatal sepsis. *Pediatric Infectious Disease Journal*.

[41] Philip AG, Mills PC (2000). Use of C-reactive protein in minimizing antibiotic exposure: experience with infants initially admitted to a well-baby nursery. *Pediatrics*.

[42] Ehl S, Gering B, Bartmann P, Högel J, Pohlandt F (1997). C-reactive protein is a useful marker for guiding duration of antibiotic therapy in suspected neonatal bacterial infection. *Pediatrics*.

[43] Jaswal RS, Kaushal RK, Goel A, Pathania K (2003). Role of C-reactive protein in deciding duration of antibiotic therapy in neonatal septicemia. *Indian Pediatrics*.

[44] Al-Zwaini EJ (2009). C-reactive protein: a useful marker for guiding duration of antibiotic therapy in suspected neonatal septicaemia?. *Eastern Mediterranean Health Journal*.

[45] Squire EN, Reich HM, Merenstein GB, Favara BE, Todd JK (1982). Criteria for the discontinuation of antibiotic therapy during presumptive treatment of suspected neonatal infection. *Pediatric Infectious Disease*.

[46] Bennet R, Eriksson M, Nord CE, Zetterstrom R (1986). Fecal bacterial microflora of newborn infants during intensive care management and treatment with five antibiotic regimens. *Pediatric Infectious Disease*.

[47] Shlaes DM, Gerding DN, John JF (1997). Society for healthcare epidemiology of america and infectious diseases society of america joint committee on the prevention of antimicrobial resistance: guidelines for the prevention of antimicrobial resistance in hospitals. *Clinical Infectious Diseases*.

[48] Michael Cotten C, Taylor S, Stoll B (2009). Prolonged duration of initial empirical antibiotic treatment is associated with increased rates of necrotizing enterocolitis and death for extremely low birth weight infants. *Pediatrics*.

[49] Clark RH, Bloom BT, Spitzer AR, Gerstmann DR (2006). Empiric use of ampicillin and cefotaxime, compared with ampicillin and gentamicin, for neonates at risk for sepsis is associated with an increased risk of neonatal death. *Pediatrics*.

[50] Cotten CM, McDonald S, Stoll B, Goldberg RN, Poole K, Benjamin DK (2006). The association of third-generation cephalosporin use and invasive candidiasis in extremely low birth-weight infants. *Pediatrics*.

[51] Benjamin DK, Stoll BJ, Fanaroff AA (2006). Neonatal candidiasis among extremely low birth weight infants: risk factors, mortality rates, and neurodevelopmental outcomes at 18 to 22 months. *Pediatrics*.

[52] Gewolb IH, Schwalbe RS, Taciak VL, Harrison TS, Panigrahi P (1999). Stool microflora in extremely low birthweight infants. *Archives of Disease in Childhood: Fetal and Neonatal Edition*.

[53] Engle WD, Jackson GL, Sendelbach D (2000). Neonatal pneumonia: comparison of 4 vs 7 days of antibiotic therapy in term and near-term infants. *Journal of Perinatology*.

[54] Chowdhary G, Dutta S, Narang A (2006). Randomized controlled trial of 7-day vs. 14-day antibiotics for neonatal sepsis. *Journal of Tropical Pediatrics*.

[55] Gathwala G, Sindwani A, Singh J, Choudhry O, Chaudhary U (2010). Ten days vs. 14 days antibiotic therapy in culture-proven neonatal sepsis. *Journal of Tropical Pediatrics*.

[56] Tunkel AR, Hartman BJ, Kaplan SL (2004). Practice guidelines for the management of bacterial meningitis. *Clinical Infectious Diseases*.

[57] Sáez-Llorens X, McCracken GH (2003). Bacterial meningitis in children. *Lancet*.

[58] Kline MW (1988). Citrobacter meningitis and brain abscess in infancy: epidemiology, pathogenesis, and treatment. *Journal of Pediatrics*.

[59] Unhanand M, Mustafa MM, McCracken GH, Nelson JD (1993). Gram-negative enteric bacillary meningitis: a twenty-one-year experience. *Journal of Pediatrics*.

[60] Karageorgopoulos DE, Valkimadi PE, Kapaskelis A, Rafailidis PI, Falagas ME (2009). Short versus long duration of antibiotic therapy for bacterial meningitis: a meta-analysis of randomised controlled trials in children. *Archives of Disease in Childhood*.

[61] Goldwater PN (2005). Cefotaxime and ceftriaxone cerebrospinal fluid levels during treatment of bacterial meningitis in children. *International Journal of Antimicrobial Agents*.

[62] Daoud AS, Batieha A, Al-Sheyyab M, Abuekteish F, Obeidat A, Mahafza T (1999). Lack of effectiveness of dexamethasone in neonatal bacterial meningitis. *European Journal of Pediatrics*.

